# Conformational flexibility and transient structure of the proline-rich domain in p53

**DOI:** 10.1016/j.bpj.2026.03.024

**Published:** 2026-03-14

**Authors:** Agnes Berggren, Michael Bakker, Hayden Fisher, Marie Skepö

**Affiliations:** 1 Division of Computational Chemistry, Department of Chemistry, Science for Life Laboratory, Lund University, P.O. Box 124, 22100 Lund, Sweden; 2 NanoLund, Lund University, P.O. Box 118, 22100 Lund, Sweden; 3 Faculty of Pharmacy in Hradec Králové, Charles University, Akademika Heyrovského 1203/8, 500 05 Hradec Králové, Czech Republic; 4 European Synchrotron Radiation Facility, Cedex 9, 38043 Grenoble, France

## Abstract

The proline-rich domain (PRD) of the tumor suppressor p53 plays a central role in modulating conformational dynamics and molecular interactions, yet its intrinsic structural behavior remains incompletely understood. Here, we combine extensive all-atom molecular dynamics simulations with biophysical validation to characterize the conformational ensemble of the p53 PRD. The domain behaves as an intrinsically disordered region, sampling a highly heterogeneous ensemble with average end-to-end distance and radius of gyration of 52.5 Å and 21.8 Å, respectively. Despite this disorder, transient local structure is prominent: unordered conformations dominate, followed by substantial polyproline II (PPII) content, with β-bends and turns linking conserved PXXP motifs. Circular dichroism and small-angle X-ray scattering experiments corroborate the largely disordered yet partially structured nature of the PRD. Ramachandran and contact analyses reveal that consecutive prolines, particularly Pro71–Pro72, impose steric constraints that stabilize locally extended conformations and restrict backbone collapse. To approximate the PRD within full-length p53, additional simulations were performed with restrained terminal distances, yielding reduced conformational variability and improved agreement with small-angle X-ray scattering data while preserving secondary-structure propensities. PPII helices emerge as particularly robust features, acting as stiff spacers linking the transactivation domain to downstream regions. Finally, simulations of clinically relevant variants reveal mutation-specific local perturbations: P72R disrupts consecutive proline rigidity and increases flexibility, whereas P82L abolishes a PXXP motif and its associated PPII helix. These results identify proline-mediated rigidity and transient PPII structure as key determinants of the dynamic conformational landscape of the p53 PRD.

## Significance

The proline-rich domain of p53 modulates its conformational dynamics and interactions. We show that consecutive prolines stabilize extended regions, and robust polyproline II helices act as structural hinges. Disease-associated variants locally disrupt these elements, altering flexibility. These results provide a molecular explanation for how proline-mediated structure regulates p53 function and how proline-rich domain mutations can impact protein dynamics, offering insight into the role of internal disordered regions in tumor suppression.

## Introduction

**Figure 1:**
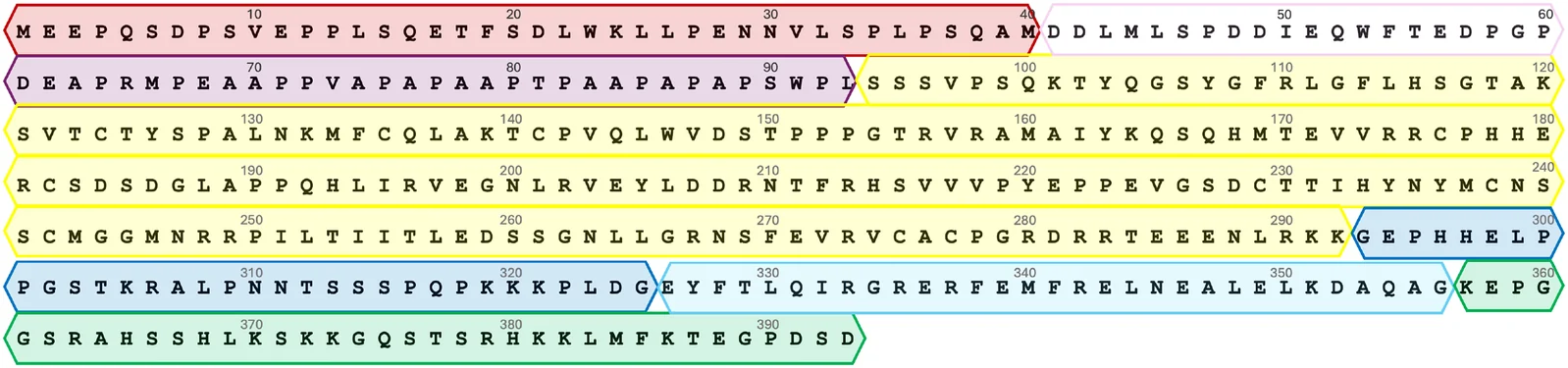
p53 sequence. Domains in order: transactivation domain 1 (TAD1, 1–40, *dark red*), transactivation domain 2 (TAD2, 40–60, *pink*), proline-rich domain (PRD, 64–92, *purple*), DNA-binding domain (DBD, 94–292, *yellow*), pretetramerization loop (PTL, 292–325, *blue*), tetramerization domain (TET, 325–356, *cyan*), and regulatory domain (REG, 356–393, *green*).

p53 is a tumor suppressor protein encoded by the *TP53* gene (1). Functioning as a tetrameric transcription factor, p53 binds DNA, actively scans for genomic damage, and initiates cellular responses such as apoptosis or growth arrest (2,3). By preventing the replication of damaged DNA, p53 plays a critical role in maintaining genomic integrity. Inactivation of p53 through deletion, mutation, or interaction with viral proteins is recognized as a key step in the development of more than 50% of human cancers (2,3). Structurally, p53 comprises several ordered and intrinsically disordered domains (see Fig. 1), including two transactivation domains (TAD1 and TAD2), a proline-rich domain (PRD), a DNA-binding domain (DBD), a pretetramerization loop (PTL), a tetramerization domain (TET), and a regulatory domain (REG) (1,2).

**Figure 2:**
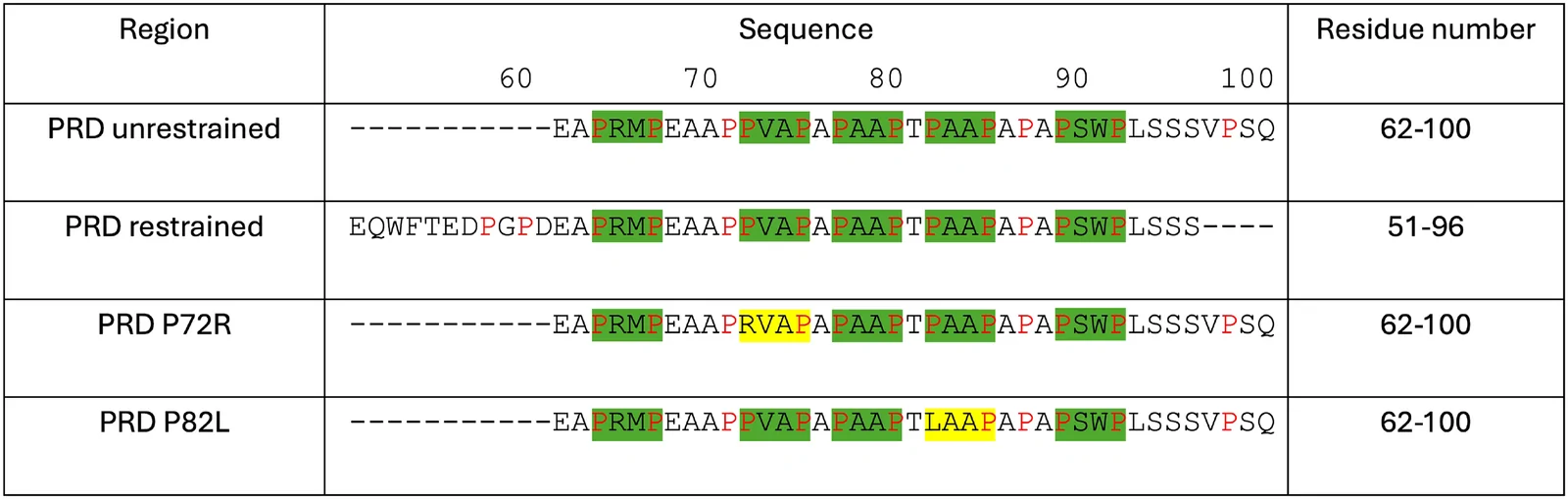
Proline-rich domain sequence. The proline-rich domain sequences chosen for the simulations. Proline residues are colored red, and all PXXP motifs (Proline-any-any-Proline) are highlighted in green. PXXP motifs where a proline residue has been mutated are highlighted in yellow. Due to two separate studies, the sequences between restrained and unrestrained simulations differ slightly. Notice, though, that we do not expect any significant effects on the overall results since the proline patterning and motif locations are preserved. Theoretical end-to-end distances from the PDB structure are 41.1 Å for the restrained model and 47.7 Å for the unrestrained sequence. Linear charge density, λ, is −0.0072 Å^−1^.

This study focuses on the PRD, the sequence of which is presented in Fig. 2. The domain derives its name from its unusually high proline content, with proline residues accounting for 33.3% of the sequence (4,5). The proline residues are evenly distributed throughout the domain, including one instance of two consecutive prolines. The PRD contains five conserved PXXP motifs (Proline-any-any-Proline), which are required for efficient p53-mediated apoptosis (3,5,6) and contribute to growth suppression (5,6). Structural studies have shown that PXXP motifs commonly adopt polyproline type II (PPII) helices—extended, left-handed secondary structures lacking intrachain hydrogen bonds. These helices frequently serve as recognition sites for SH3 (Src homology 3) domains, which are common in signal-transducing proteins (5,7,8,9).

Proline is unique among amino acids in its ability to adopt both *cis* and *trans* peptide-bond conformations, whereas other residues overwhelmingly favor the *trans* state (10,11,12). Formation of PPII structure requires the proline residues to be in the *trans* peptide-bond conformation (13). However, depending on the local sequence context, approximately 5%–10% of proline residues in disordered proteins populate the *cis* conformation (10,12,14). This behavior arises from the reduced energetic penalty and lower entropic cost of isomerization associated with proline’s cyclic side chain (10,11). Peptidyl-prolyl isomerases such as PIN1 catalyze this interconversion by transiently disrupting the partial double-bond character of the peptide bond (11).

Despite containing these transient structures, the PRD is intrinsically disordered, meaning it samples a broad ensemble of transient and structurally distinct conformations, which complicates its characterization (15,16). Most experimental techniques provide time- or ensemble-averaged observables, such that the resulting structural models represent averages over many conformations rather than any specific structure adopted by the protein at a given time (16). This averaging obscures the underlying conformational heterogeneity that defines intrinsically disordered proteins (IDPs). Molecular dynamics (MD) simulations provide a powerful complementary approach by enabling direct observation of protein motions over time (16). However, in the absence of enzymatic catalysis, *cis*-*trans* isomerization occurs on timescales of seconds to minutes, far exceeding those accessible to conventional MD simulations (10,11). Consequently, MD studies typically restrain prolines in either the *cis* or *trans* state and combine results post hoc, a strategy that becomes impractical for proline-rich sequences due to the exponential growth in possible conformational combinations (10).

Furthermore, the PRD is typically simulated as an IDP, characterized by high conformational flexibility. In contrast, intrinsically disordered regions (IDRs) are internal segments tethered to globular domains at both termini. Although this arrangement permits relative motion of the adjacent domains, it also restricts the conformational space accessible to the IDR itself (1,17). The influence of such terminal constraints has been investigated in previous studies of other p53 domains, where incorporating restraints led to improved agreement between simulations and experimental data (1,17).

Among the many missense mutations reported for p53, 53 mutations have been identified within the PRD, with alanine-proline motifs being particularly frequently affected (18). Several mutation hotspots occur at positions 72, 73, 82, 84, 89, and 98 (7,8,9). All of these residues are located within PXXP motifs, and four positions, 72, 82, 89, and 98, are prolines. Two mutations at these positions, P72R and P82L, are investigated in this study (see Fig. 2).

P72R represents a polymorphism with geographical variation: proline is more prevalent in individuals of African descent, whereas arginine is more commonly observed in Caucasian populations (8,9). The PRD containing proline at position 72 is referred to here as the wild-type (WT). The P72R variant has been reported to exhibit subtle structural differences, including altered migration behavior in SDS-PAGE experiments (9,19). Although both variants are functional, differences have been observed in cancer susceptibility (8) and in the efficiency of apoptosis induction and cell-cycle arrest (9). Notably, the presence of an arginine followed by three or more residues upstream of a PXXP motif is known to enhance SH3 domain binding, which may help explain the persistence of this variant despite the loss of one PXXP motif (8).

In contrast, the P82L mutation has been associated with impaired p53 function. Previous studies report that P82L correlates with reduced p300-mediated acetylation (8) and diminished CHK-2–mediated phosphorylation (7,9,20,21) within the transactivation domain, posttranslational modifications that are important for transcriptional activation. In the WT PRD, PIN1 is known to bind the Thr81–Pro82 motif in response to DNA damage and to catalyze the *cis*-*trans* isomerization of Pro82, a process proposed to facilitate subsequent phosphorylation of Ser20 in the TAD (7,9,20,21). Nuclear magnetic resonance (NMR) studies indicate that most proline residues in the PRD predominantly adopt the *trans* conformation, whereas a minor *cis* population is observed specifically for Pro82, consistent with PIN1 activity (7). Notably, this *cis* population is not detected in mutants such as P82L (7). Collectively, these observations support a model in which the P82L mutation perturbs PIN1-dependent regulatory mechanisms, potentially contributing to compromised p53 responses under conditions of cellular stress.

This study aims to deepen our understanding of the structural behavior of the p53 PRD and, more broadly, the simulation of proline-rich IDRs. A central objective is to assess how the PRD can be accurately represented using MD simulations by modeling it both as an unconstrained IDP and as an internally constrained IDR. We further examine how the proline-rich sequence and conserved PXXP motifs influence transient secondary-structure formation and overall conformational behavior by comparing the WT PRD with the P72R and P82L variants. These two mutations, one functional polymorphism and one cancer-associated mutation, serve as representative examples to elucidate how local proline substitutions modulate the conformational landscape of the PRD.

## Materials and methods

### Atomistic molecular dynamics simulations

Fully atomistic MD simulations were conducted using the GROMACS package version 2024 (22), employing the AMBER99SB-ILDN force field and TIP4P-D water model (23,24). This force field has been used in previous simulations of intrinsically disordered p53 domains (1,17) and generates conformational ensembles with radius of gyration ($R_{g}$) and secondary structure distributions comparable to other IDP-capable force fields for proline-rich domains (25). The peptide was simulated in its zwitterionic state at natural pH, a neutral net charge, and with a NaCl concentration of both 10 mM and 150 mM for unstrained simulations and 10 mM for restrained and mutant simulations. A reduced salt concentration (10 mM) was used to enable clearer assessment of electrostatic effects while maintaining consistency across simulations. An additional unrestrained simulation at physiological ionic strength (150 mM) was included to assess the robustness of the observed behavior under biologically relevant conditions. A rhombic dodecahedron box was used, and periodic boundary conditions were applied in all three dimensions. Starting conformations of both WT and mutant PRD peptides were generated using Avogadro version 1.2.0 (26). For unrestrained peptides, the minimum distance between the peptide and the box edge was set to 1 nm. For restrained systems, the distance was increased to 2 nm, and restraints were applied to the first and last residues by including the *freeze_grps* command in the MDP file to lock their *x,y,z* positions (1,17) with distances of around 1, 2, 3, 4, and 5 nm.

GROMACS’ leap-frog algorithm was implemented with a time step of 2 fs. Short-range interactions were handled using a Verlet cutoff scheme with a 12-Å cutoff. Long-range electrostatics were treated using the particle-mesh Ewald method (27). All bonds involving hydrogen atoms were constrained using the LINCS algorithm (28). Temperature and pressure were maintained at 298 K and 1 bar, respectively, using the Nose-Hoover thermostat (29) and Parrinello-Rahman barostat (30). Energy minimization was carried out using the steepest descent algorithm.

For each simulation, five replicates were made. The total simulation run time was 15.6 μs for the unrestrained low salt concentration simulations, 15 μs for the unrestrained high salt concentration simulations, 13 μs for P72R, and 10 μs for P82L. Each system underwent equilibration for 0.5 ns under constant temperature (NVT), followed by 0.5 ns at constant pressure and temperature (NPT), and an additional 1 ns in the NPT ensemble. For restrained peptides, five replicates with a total simulation run time of 5.5 μs were run. Equilibration was performed for 0.5 ns and 1 ns under NVT and NPT conditions, respectively.

An additional five independent 2-μs trajectories of the p53 PRD were generated, in which Pro82 was constrained to adopt the *cis* conformation rather than its original *trans* state, but otherwise following the same procedure as for the WT p53 PRD simulations. The isomerization of Pro82 was introduced using ChimeraX version 1.9 (31) before simulation.

### Analysis of trajectories

To avoid bias from initial peptide contraction, the first 200 ns of each unrestrained simulation and the first 100 ns of each restrained simulation were removed using *gmx trjconv*. The five replicates for each simulation were then concatenated using *gmx trjcat* to create a single trajectory for analysis.

$\left(R_{g}\right)$, $R_{ee}$, and their distributions were calculated using *gmx polystat* and *gmx analyze*. Secondary structure was analyzed using the Define Secondary Structure of Proteins (DSSP) algorithm via *gmx dssp*, and Ramachandran plots were generated by *gmx rama*. Minimum distance maps showing either the minimum interresidue distance or hydrogen-bonding patterns were generated using a homemade Python script in combination with *gmx pairdist* or *gmx hbond*, respectively. Solvent-accessible surface area was calculated using *gmx sasa*.

Theoretical small-angle X-ray scattering (SAXS) profiles were computed using CRYSOL version 3.0 from the ATSAS package version 3.2.1 (32,33). Further decomposition of the conformational ensemble (CoE) based on the SAXS was done by separating the trajectory into groups based on $R_{g}$ using a custom script (34). Snapshots from the simulations were made using ChimeraX version 1.9 (31). Root-mean-square deviation and root-mean-square fluctuation as well as phi (ϕ), psi (ψ), and omega (ω) angles in the peptide chain were calculated with the Python package *MDTraj* (35). Theoretical NMR chemical shifts were generated using the SPARTA+ web server (36) and compared with available NMR data from the Biological Magnetic Resonance Data Bank (BMRB: 51,984 (7) and 50,960 (37)).

### Circular dichroism

The synthesized peptide powder (purity 95%) was obtained from Chemtronica, Stockholm. The peptide was dissolved in a buffer of NaF (10 and 150 mM, respectively) and 20 mM sodium phosphate (pH 7.0). The protein solution was dialyzed using dialysis cassettes (Slide-A-${\text{Lyzer}}^{TM}$, 2K MWCO), and concentrations were measured using a Nanodrop 2000 instrument at 280 nm. Measurements on the WT protein domain were performed at the ASTRID2 beamline at the Center for Storage Ring Facilities in Aarhus (ISA), Denmark. These spectra were produced for both salt concentrations at protein concentrations of 0.2 mg/mL, 1 mg/mL, and the stock solution. Circular dichroism (CD) spectra for WT at 10° C and mutants P72R and P82L were measured using a Jasco J-715 spectropolarimeter. The CD signal is converted to Δϵ for analysis using Eq. 1:

(1) $$\Delta \epsilon \left(M^{-1}cm^{-1}\right)=\frac{CD\left(mdeg\right)\cdot MRW\left(Da\right)}{32980\cdot l\left(cm\right)\cdot c\left(g/L\right)}$$

where *CD* is the measured CD signal, *l* is the pathlength of the cell, *c* is the protein concentration of the sample, and *MRW* is the mean residue weight. The latter is calculated from Eq. 2:

(2) $$MRW=\frac{MW}{\text{Sequence}\;\text{length}-1}$$

where *MW* is the molecular weight of the protein. Because PPII helices are difficult to detect directly, a qualitative approach was used to estimate their content (38). This method exploits the thermal destabilization of PPII by monitoring changes in the CD signal at 228 nm (39). In CD spectra, PPII exhibits a maximum at 228 nm, a minimum at 205 nm, and an isodichroic point at 211 nm (38,39,40). As the 205-nm minimum overlaps with the signal of disordered proteins, analysis focuses on the temperature-dependent changes at 228 nm, which are expected to be linear and reversible (39,40).

### Small-angle X-ray scattering

The synthesized peptide powder (purity 95%) was obtained from Chemtronica, Stockholm. The peptide was dissolved in a buffer of NaCl (10 and 150 mM, respectively) and 20 mM TRIS (pH 7.0). The protein solution was dialyzed using dialysis cassettes (Slide-A-${\text{Lyzer}}^{TM}$, 2K MWCO) and concentrations were measured using a Nanodrop 2000 instrument at 280 nm. SAXS measurements were performed at the BM29 BioSAXS beamline at the European Synchrotron Radiation Facility (ESRF) in Grenoble, France. All measurements were performed at an energy of 12.5 keV and a temperature of 20°C with a q-range of 0.00776–0.495 Å^−1^. Ten frames were collected for each sample. Frames affected by radiation damage were discarded before averaging, and the buffer was subtracted from the sample.

The experimental data were analyzed using PRIMUS, and re-binning was performed with DATREGRID, both part of the ATSAS package (32). To enable quantitative comparison between experimental and simulated SAXS profiles, the scattering intensities were normalized to $I_{0}$ before calculation of a normalized chi-square metric, $\chi_{\text{norm}}^{2}$, defined as

(3) $$\chi_{\text{norm}}^{2}=\overset{N}{\underset{i=1}{\sum }}\frac{{\left(E_{i}-S_{i}\right)}^{2}}{S_{i}^{2}},$$

where $E_{i}$ and $S_{i}$ denote the experimental and simulated intensities at data point i, respectively. This metric was used for internal comparison between different simulation ensembles and does not represent a statistically rigorous goodness of fit. Low values indicate close agreement between profiles, whereas increasing values signify progressively larger discrepancies in the structural features encoded by the scattering data. For visualization purposes, the SAXS profiles are shown normalized in the high-q region to facilitate comparison of differences at low q.

All data were deposited in the SASBDB with the accession codes SASDY42, SASDY52, SASDY62, SASDY72, SASDY82, and SASDY92.

## Results and discussion

### The p53 PRD samples a broad CoE with persistent local structure

**Figure 3:**
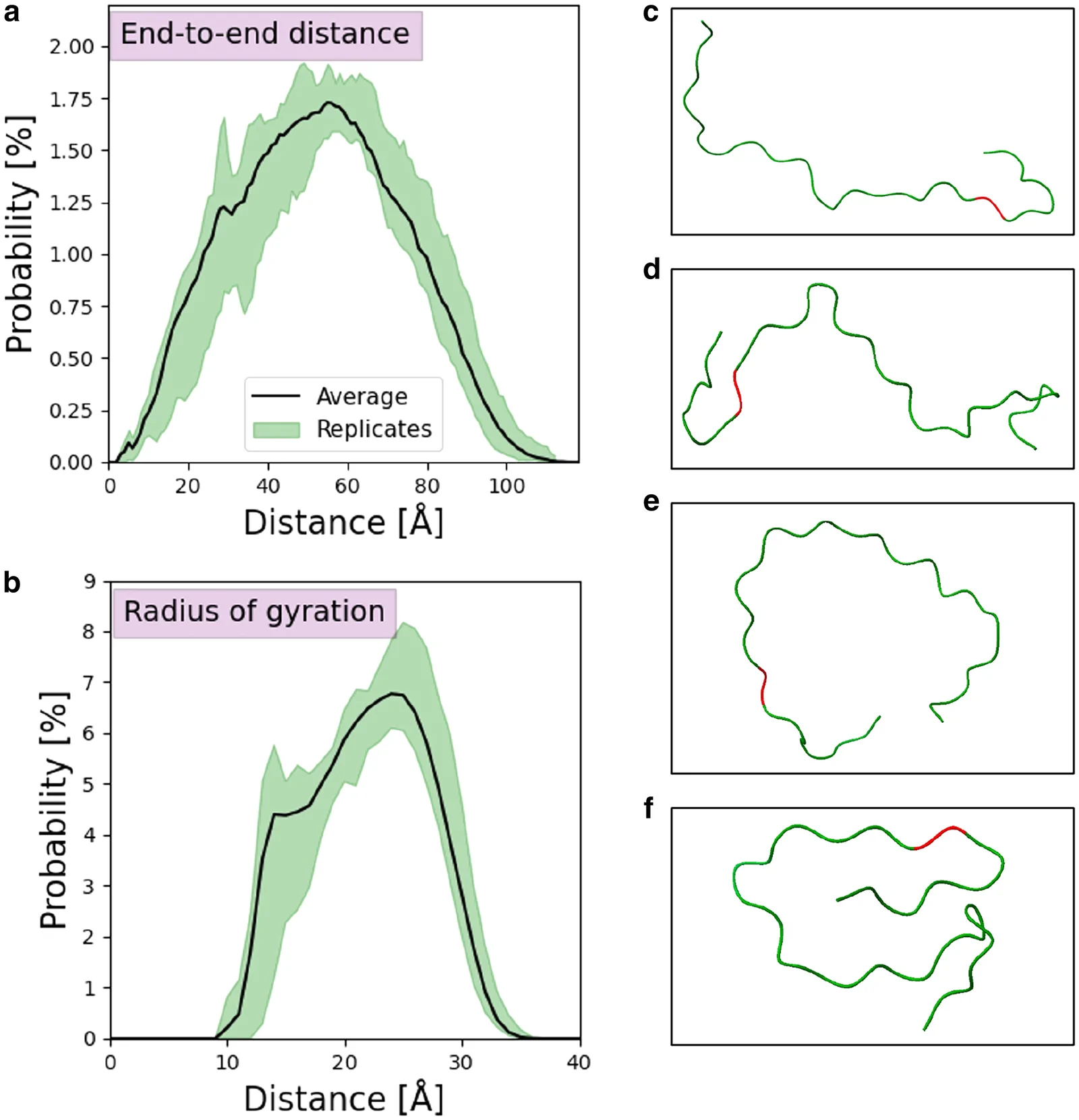
End-to-end distance and radius of gyration analysis. Figure shows the ensemble distribution of the end-to-end distance (*a*) and radius of gyration, $R_{g}$, (*b*) for each frame during the unrestrained WT simulations. The shaded area shows the deviation from the average for the five separate replicates. The black line is the average. (*c*–*f*) Snapshots from simulation represented by conformations ranging from extended to compact. The red residues are the consecutive Pro71-Pro72.

The p53 PRD was simulated as an IDP, modeled as an isolated, unrestrained polypeptide chain in solution. Across five independent all-atom MD trajectories, the PRD samples a wide range of conformations, consistent with its intrinsically disordered character. Time series of the end-to-end distance ($\left\langle R_{ee}\right\rangle$) and radius of gyration ($\left\langle R_{g}\right\rangle$) reveal large fluctuations within each trajectory and substantial overlap between replicates (Fig. 1, *a*). Averaged over all simulations, $\left\langle R_{ee}\right\rangle$ = 52.5 Å and $\left\langle R_{g}\right\rangle$ = 21.8 Å. Ensemble distributions of $R_{ee}$ and $R_{g}$ (Fig. 3, *a* and *b*) confirm that the PRD populates both compact and highly extended conformations, with $R_{g}$ spanning approximately 9–36 Å and $R_{ee}$ values extending over ∼110 Å. These distributions are characteristic of disordered polypeptides lacking a single dominant fold (15), (16). Given this pronounced heterogeneity, subsequent analyses focus not on individual conformations but on ensemble-averaged properties and systematic trends.

### Persistent polyproline II structure dominates the local conformational landscape

**Figure 4:**
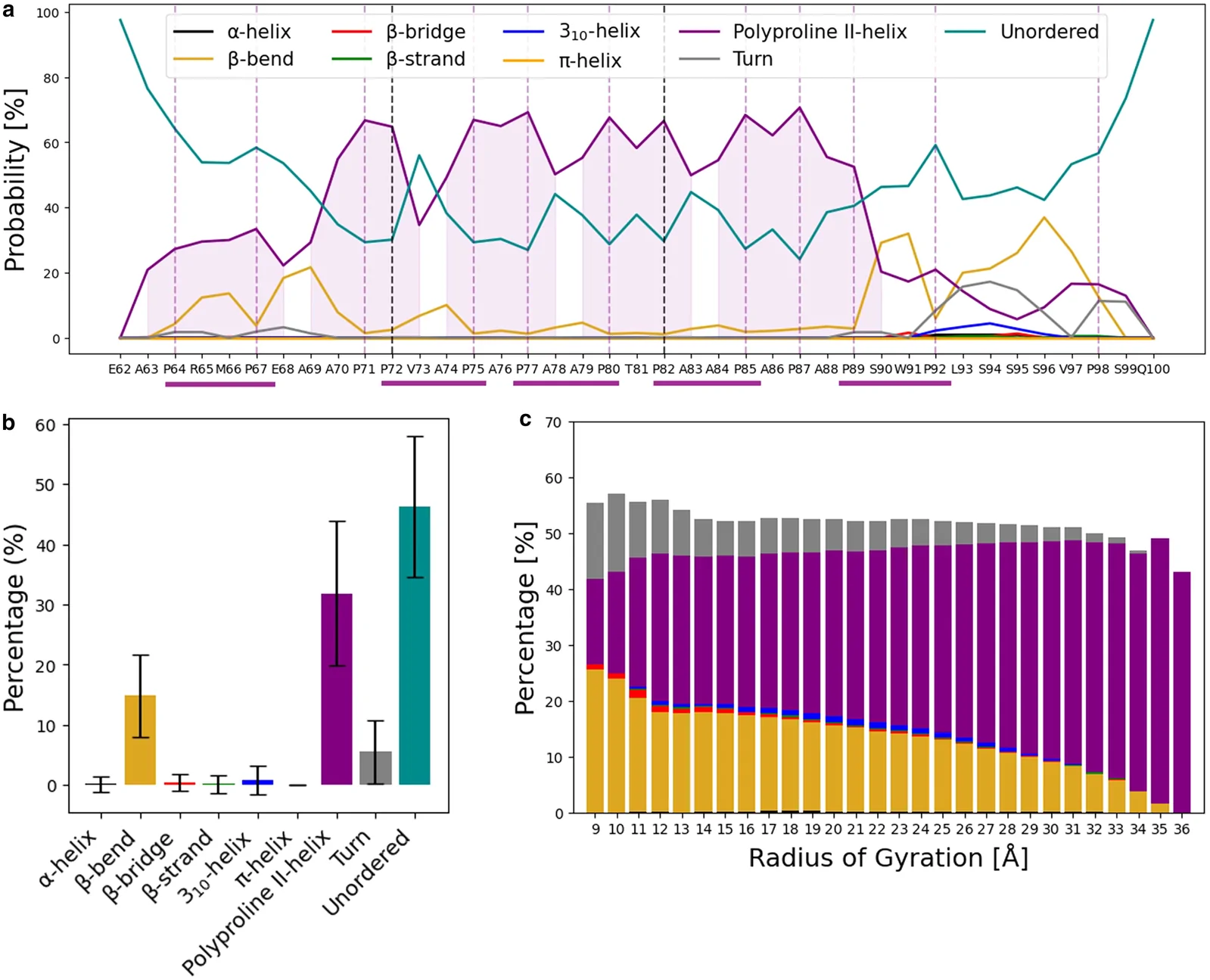
DSSP analysis of the unrestrained WT simulations. (*a*) Residue-wise secondary structure probability. The PXXP (Proline-any-any-Proline) motifs are underlined, and all proline residues are marked with vertical dashed lines. The black dashed lines are Pro72 and Pro82. The five polyproline II (PPII) helical peaks are shaded for emphasis. (*b*) Total average secondary structure probability of PRD. Error bars represent the standard deviation. (*c*) Average transient secondary structure composition per radius of gyration group.

To relate global chain dimensions to local structural preferences, the CoE was decomposed into subsets based on $R_{g}$ (Fig. 3, *c*–*e*). Secondary structure propensities were quantified using DSSP and averaged over all trajectories, with uncertainties estimated from the five independent simulations. The PRD is predominantly disordered, with unordered conformations accounting for 45.1% ± 11.6% of the ensemble (Fig. 4, *b*). However, PPII helices constitute a substantial fraction (31.1% ± 11.9%), followed by β-bends (14.4% ± 5.2%) and turns (5.3% ± 1.3%). Stable α-helical or β-sheet structure is negligible.

PPII propensity is not uniformly distributed along the sequence. Five local maxima are observed, each aligning within approximately three residues of a PXXP motif (Fig. 4, *a*). Notably, shallow dips in PPII propensity occur directly at the PXXP motifs themselves. Closer inspection indicates that the strongest enrichment often coincides with PP or PXP elements embedded within or adjacent to these motifs, rather than peaking strictly at the central residues of the canonical PXXP sequence. Although isolated PXXP motifs are commonly associated with high PPII propensity, PPII structure is also strongly favored in other proline-rich motifs, including polyproline stretches and PXP repeats (5,7,8,9,41). In the present sequence context, where multiple proline-rich motifs occur in close proximity, the PPII signal appears to extend across short proline clusters rather than being confined to individual consensus motifs, whereas still spanning much of the domain. In contrast, β-bends and turns preferentially populate the linker regions between proline-rich segments, suggesting a modular organization in which transient PPII-like stretches are interspersed with more flexible hinge regions. This pattern highlights the importance of proline patterning, rather than simply proline abundance, in shaping the local conformational landscape.

### Structural determinants underlying PPII stabilization

Analysis of backbone dihedral angle distributions reveals a pronounced population in the PPII region of the Ramachandran plot (Fig. S2, *a*), corroborating the DSSP results. Minimum interresidue distance maps (Fig. S2, *b*) highlight a distinctive feature centered on residues 71–72, the only pair of consecutive prolines in the sequence. These adjacent prolines impose strong steric constraints that limit backbone collapse and favor locally extended conformations (Fig. 3, *c*–*f*). Hydrogen-bond analysis (Fig. 2, *c*) reveals a low overall hydrogen-bonding propensity, consistent with the scarcity of backbone amide hydrogens in proline-rich sequences. Localized hydrogen-bonding features coincide with β-bend regions identified by DSSP, indicating transient stabilization of these motifs. Inclusion of *cis*-proline conformations would be expected to increase the frequency of such local hydrogen-bonded turns while reducing the persistence of extended PPII segments, without eliminating them.

### Coupling between chain extension and PPII enrichment

When the ensemble is stratified by $R_{g}$, a clear dependence of secondary structure composition on global dimensions emerges (Fig. 4, *c*). Compact conformations (low $R_{g}$) are enriched in β-bends and turns, whereas extended conformations show a pronounced increase in PPII content. Specifically, the PPII fraction increases from 20% in the most compact subsets to over 40% in the most extended subsets, while β-bend content decreases from 25% to nearly zero. These trends indicate that extended PRD conformations are stabilized by locally extended backbone geometries characteristic of PPII helices, whereas compaction is associated with increased backbone bending.

### Impact of *cis*-*trans* proline isomerization: Limitation and assessment

All simulations presented here were performed using standard all-atom force fields in which proline residues remain in the *trans* configuration throughout the simulation timescale (10,11). *Cis*-*trans* isomerization of the peptide bond preceding proline occurs on timescales of seconds to minutes and is therefore not sampled in conventional MD simulations without enhanced sampling methods. Experimental and computational studies indicate that *cis*-proline conformations populate approximately 5%–10% of the equilibrium ensemble in proline-rich IDRs (10,12,14).

To assess the structural impact of this limitation, simulations of the WT PRD with Pro82 in the *cis* conformation were additionally performed to complement the experimental observations made in this paper and in previous studies using NMR (7). These simulations yielded an $R_{g}$ of approximately 19.2 Å, which is about 2.6 Å smaller than that of the all-*trans* conformation. This reduction is expected, as the *cis* conformation introduces a kink in the protein backbone, allowing the chain to adopt a more compact ensemble. Analysis of secondary structure using DSSP for this *cis*-Pro82 trajectory shows no detectable PPII probability at the affected residue (Fig. 16, *a*). Notably, the β-bend probability increases to nearly 100%, whereas the disorder probability is almost completely suppressed. In a physiological context, the effective behavior is likely to lie between these extremes, consistent with an estimated *cis* population of 5%–10% reported for proline residues in disordered proteins.

### Consistency with circular dichroism spectroscopy: Qualitative agreement

**Figure 5:**
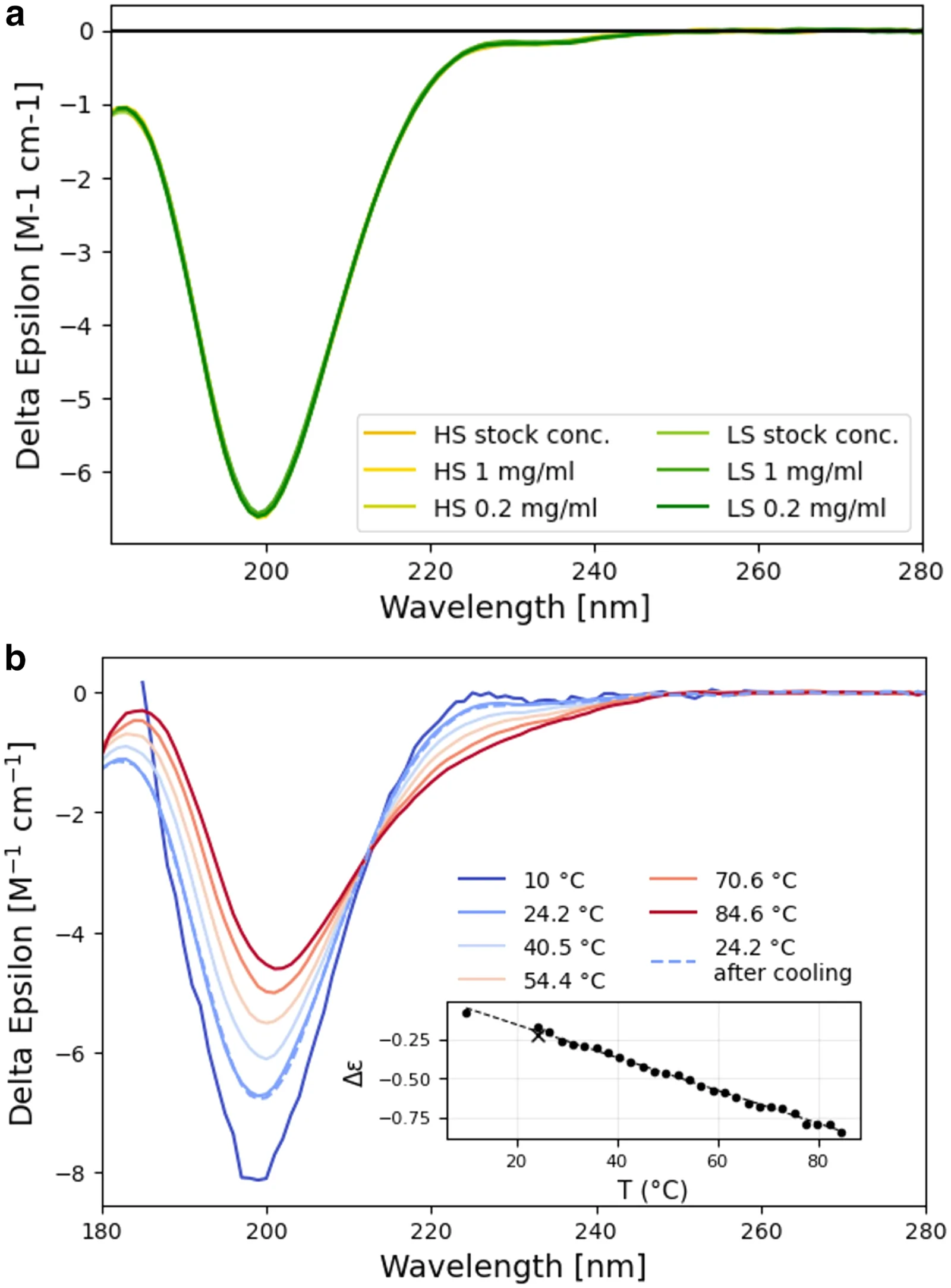
CD analysis and temperature scan. (*a*) Δϵ for the WT protein measured at different salt concentrations and protein concentrations. Stock concentrations were 8.6 mg ${\text{ml}}^{-1}$ for low salt (LS, 10 mM) and 8.9 mg ${\text{ml}}^{-1}$ for high salt (HS, 150 mM). The experimental CD spectra overlap across conditions. (*b*) Temperature-dependent CD scan of the WT protein at a concentration of 1 mg ${\text{ml}}^{-1}$. Δϵ was monitored at a wavelength of 228 nm and is shown in the inset. The linear trend yields $R^{2}=0.995$. The cross indicates the Δϵ value after cooling from the highest temperature. The series of spectra from 24.2 to 84.6 °C was measured at the ASTRID2 beamline at the Center for Storage Ring Facilities in Aarhus (ISA), Denmark. The CD spectrum of the WT protein at 10 °C was measured using a Jasco J-715 spectropolarimeter.

Experimental CD spectra of the PRD, acquired at low (10 mM NaCl) and high (150 mM NaCl) ionic strength, are invariant with respect to protein concentration and salt conditions (Fig. 5, *a*), indicating a stable CoE. The spectra are characteristic of a largely disordered protein, with a strong negative band below 200 nm and no clear signatures of stable α-helical or β-sheet structure (15).

CD spectroscopy, however, cannot unambiguously resolve PPII helices, as their spectral features overlap substantially with those of unordered and turn-like conformations (38). Moreover, commonly used CD deconvolution methods do not include a dedicated PPII basis set. As a result, PPII-like conformations are redistributed among helical, turn, and unordered categories, precluding direct quantitative comparison between DSSP-derived secondary structure populations and CD-derived fractions. For this reason, simulated CD spectra and CD deconvolution results are not used here to quantitatively validate the simulated ensembles. In particular, theoretical CD spectra generated from the simulated structures exhibit enhanced signal in spectral regions often associated with β-rich or helical structure, in clear disagreement with experiment. This discrepancy likely reflects both the absence of *cis*-proline conformations in the simulations and the inability of available CD basis sets to faithfully represent PPII helices in IDPs.

Accordingly, agreement between simulation and experiment is interpreted qualitatively rather than quantitatively. Although CD data alone cannot determine PPII content, the presence of PPII helices in the PRD is supported by DSSP analysis, Ramachandran distributions, sequence composition, motif analysis, and prior experimental and computational studies of proline-rich IDPs (5,39). Direct experimental assessment of PPII structure was performed by analyzing Δϵ at 228 nm in combination with temperature-dependent measurements, which exploit the reduced thermal stability of PPII helices (Fig. 5, *b*) (39). The CD spectra show characteristic differences at 205 nm and 228 nm, with an isodichroic point at approximately 211 nm. This behavior is consistent with PPII structure, which is destabilized upon heating, leading to a progressive loss of its spectral features (38,39,40). Monitoring the temperature dependence of Δϵ reveals a linear and reversible response, in agreement with previous experimental studies of proline-rich IDPs (39).

### Comparison with SAXS experiments: Ensemble dimensions and compaction

**Figure 6:**
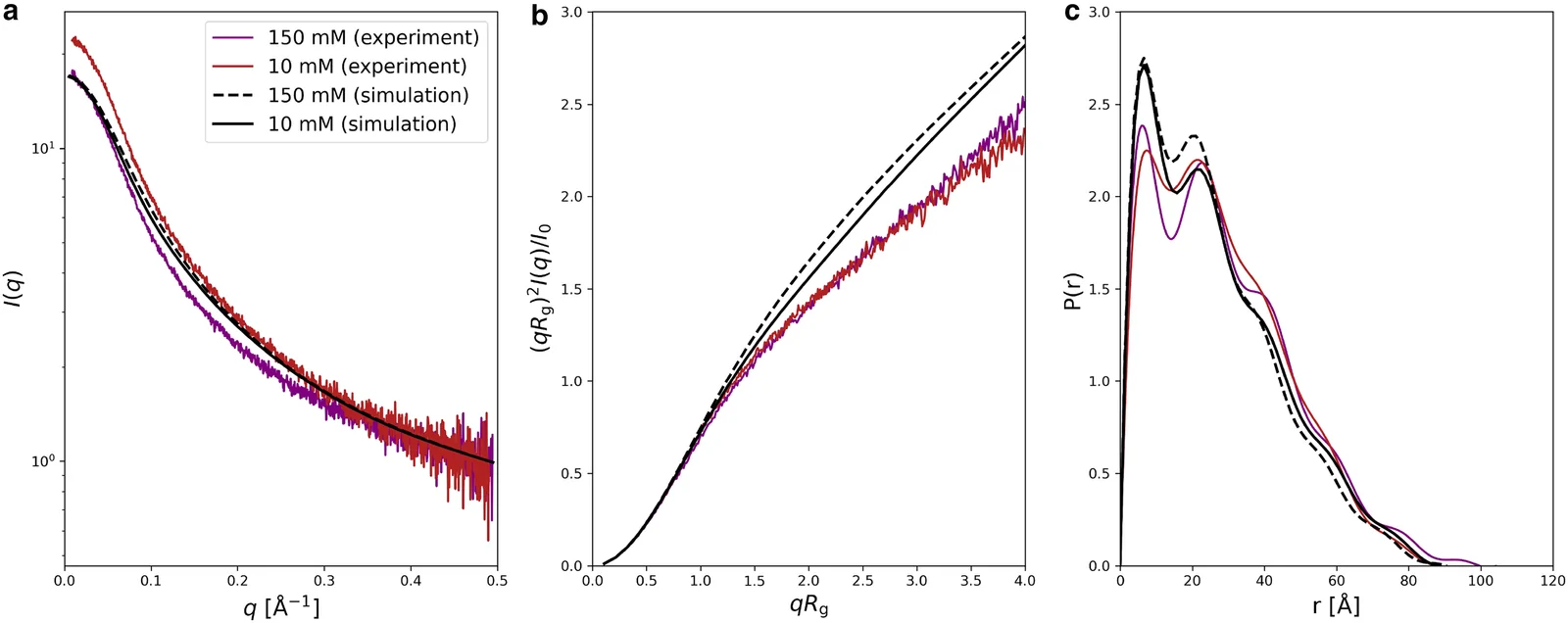
Small-angle x-ray scattering. (*a*) Form factor comparing the scattering vector to the normalized intensity, (*b*) Kratky plot, and (*c*) pair distance distribution function, P(r), for experimental and theoretical small-angle x-ray scattering results. This was measured for high (150 mM) and low (10 mM) salt concentration.

Simulated SAXS profiles were compared with experimental data collected at low and high salt concentrations (Fig. 6, *a*–*c,* Table 1). Good overall agreement is obtained, with $\chi_{\text{norm}}^{2}$ values of 0.373 (high salt) and 0.397 (low salt). Kratky representations reveal that both experiment and simulation correspond to extended, nonglobular conformations. However, the simulated ensembles appear slightly more extended than the experimental data, with similar results for the two salt concentrations. One likely contributor to this difference is the absence of *cis*-proline conformations in the simulations, which are known to promote compaction in proline-rich peptides (10). In addition, SAXS measurements are sensitive to hydration layers and excluded-volume effects that are not fully captured in standard forward models, potentially contributing to discrepancies between simulated and experimental scattering profiles. Pair distance distribution functions, P(r), show similar overall shapes and comparable $D_{\max}$ values (90–100 Å) in the experiment and simulation, supporting the conclusion that the simulations reproduce the PRD’s global dimensions within expected uncertainties.

**Table 1:** Small-Angle X-Ray Scattering Comparison**:** Radius of Gyration, $R_{g}$, and Maximum Distance Within Protein, $D_{\max}$, for the Proline-Rich Domain of p53 from Experimental SAXS Data and from the Simulations

	HS Exp.	LS Exp.	HS Sim.	LS Sim.
$R_{g}$ – Guinier (Å)	22.5 ± 0.1	21.9 ± 0.1	20.9 ± 0.2	21.9 ± 0.2
$R_{g}$ – P(r) (Å)	24.0 ± 0.2	23.4 ± 0.2	21.9 ± 0.2	22.9 ± 0.2
$D_{\max}$ (Å)	104.2 ± 0.5	88.5 ± 0.5	90.6 ± 0.5	88.0 ± 0.5

Radius of gyration, *R_g_*, was determined using both the Guinier plot and pair distribution function. This was calculated for high (HS, 150 mM) and low (LS, 10 mM) salt concentration.

### Modeling the PRD as an internal disordered region using end-to-end restraints

**Figure 7:**
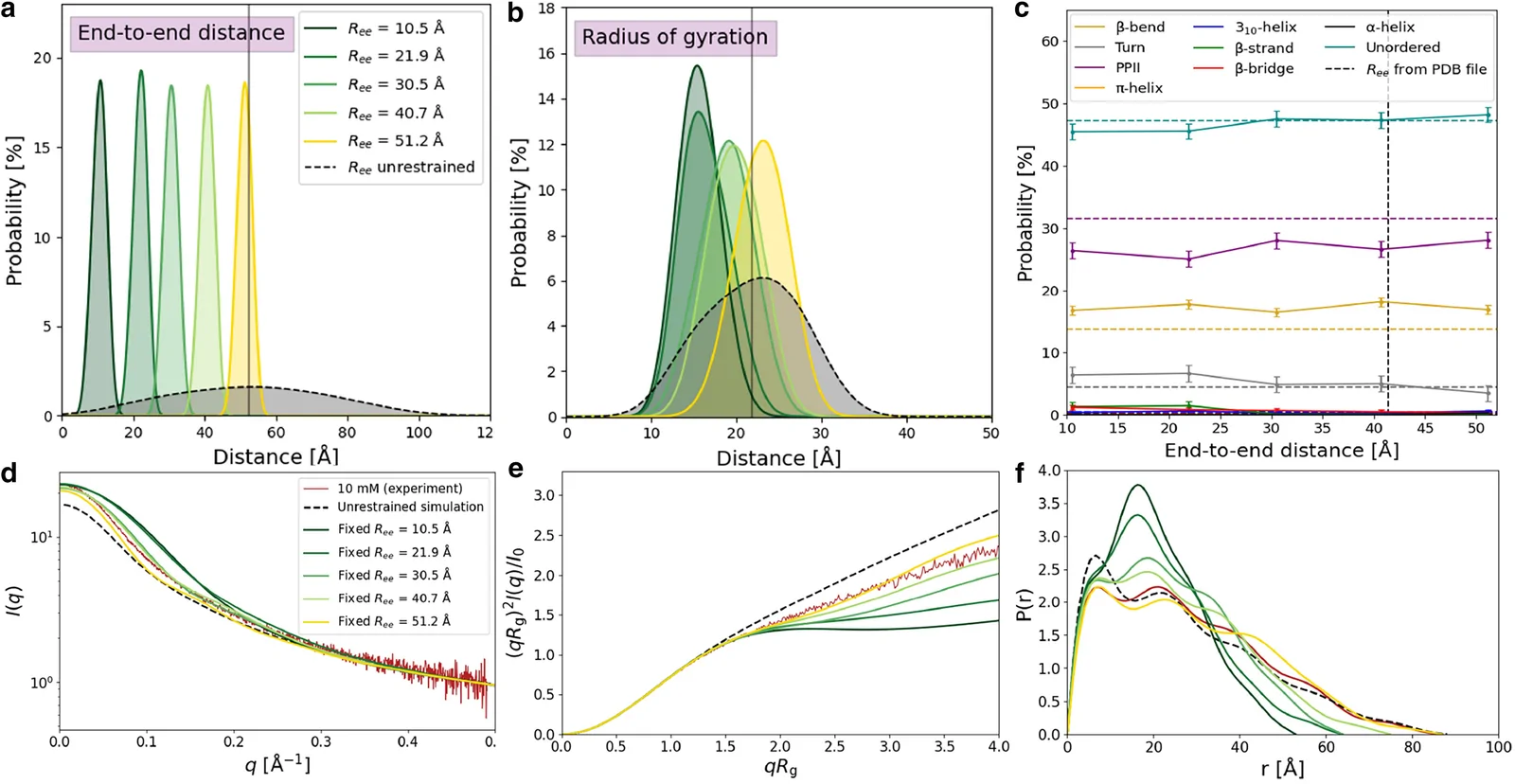
Analysis of intrinsically disordered regions with restrained end-to-end distance. (*a*) End-to-end distance $\left(R_{ee}\right)$ and (*b*) radius of gyration (*R*_*g*_) for five separate simulations with fixed $R_{ee}$ and the unrestrained simulation. $R_{ee}=52.5$ Å for the unrestrained simulations. The horizontal lines are the average $R_{g}$ and $R_{ee}$ for the unrestrained simulations. (*c*) The average secondary structure probability for trajectories with different fixed $R_{ee}$. Dashed horizontal lines show the average secondary structure probability for the unrestrained simulation. The dashed vertical line is the $R_{ee}$ of the chosen sequence from the available PDB structure (8f2i). Error bars show the standard deviation between the averages from the different trajectories. (*d*) Normalized intensity plot, (*e*) Kratky plot, and (*f*) pair distance distribution function, P(r). This was performed at low salt concentration (10 mM NaCl).

To approximate the PRD as an IDR constrained by adjacent domains in the full-length p53 protein, additional simulations were performed with restrained end-to-end distances ($R_{ee}$ = 10–50 Å). This range includes the experimental $R_{ee}$ value of 41.4 Å observed in a cryo-EM structure of p53 (PDB: 8F2I). Restrained simulations exhibit narrower $R_{ee}$ distributions and reduced conformational variability, whereas $R_{g}$ distributions partially overlap between neighboring restraint values (Fig. 7, *a* and *b*). Secondary structure analysis reveals only modest differences relative to the unrestrained ensemble, with slightly increased PPII content and reduced β-bend populations due to exclusion of the most compact conformations (Fig. 7, *c*). Importantly, the overall secondary structure fingerprint of the PRD is preserved, indicating that PPII motifs are robust to global spatial constraints.

Restrained simulations with $R_{ee}$ 40–50 Å show improved agreement with experimental SAXS profiles (Fig. 7, *d*–*f*, Table 2). This improved agreement likely reflects the fact that, in vivo and in experimental constructs, the PRD does not behave as a fully isolated chain but experiences effective spatial constraints imposed by neighboring domains. Such constraints may partially compensate for the absence of *cis*-proline conformations in the simulations, yielding ensembles that more closely resemble experimental scattering data.

**Table 2:** Small-Angle X-Ray Scattering Comparison, $\chi_{\text{norm}}^{2}$, Calculated for Restrained and Unrestrained Simulations

Simulations	LS
Unrestrained	0.397
$R_{ee}$ = 10.5 Å	1.051
$R_{ee}$ = 21.9 Å	0.820
$R_{ee}$ = 30.5 Å	0.199
$R_{ee}$ = 40.7 Å	0.133
$R_{ee}$ = 51.2 Å	0.056

Restrained simulations have a fixed end-to-end distance, $R_{ee}$. $R_{ee}=52.5$ Å for the unrestrained simulations. They are compared with experimental small-angle x-ray scattering data at low salt (LS, 10 mM) concentration. The data were normalized at the lowest q-value for easy comparison to simulated data.

### Local structural effects of P72R and P82L variants

**Figure 8:**
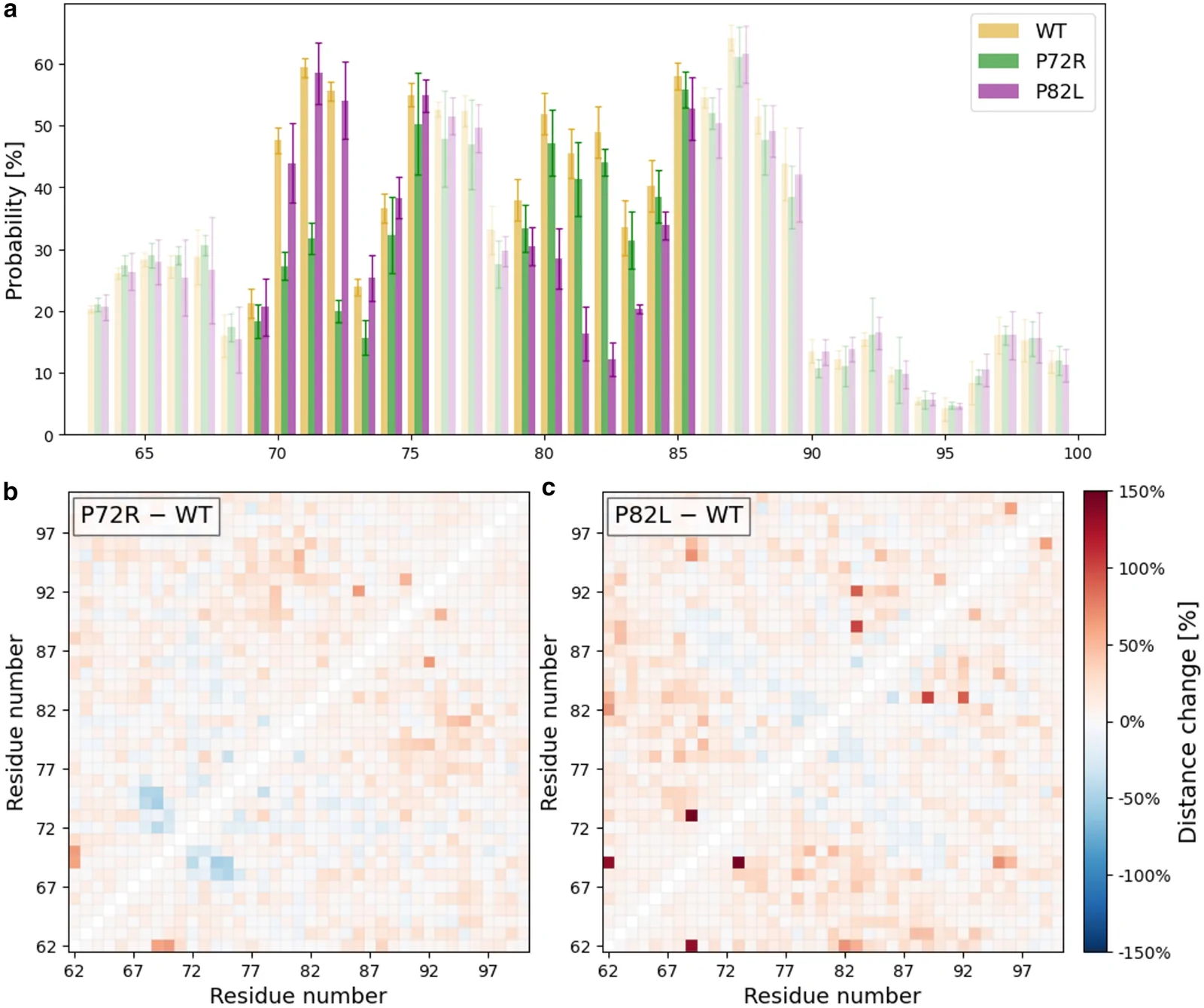
Secondary structure and minimum distance maps. (*a*) Residue-wise polyproline II-helix probability of two mutated variants P72R and P82L as well as wild-type (WT) proline-rich domain. Error bars show the standard deviation of the five replicates. Minimum distance maps show the relative difference in minimum distance between any two residue pairs for the variants (*b*) P72R and (*c*) P82L compared with WT.

Simulations of the P72R and P82L variants reveal global dimensions similar to those of the WT PRD, with only a slight shift toward more compact conformations. In contrast, local structural properties are clearly perturbed. DSSP analysis reveals a pronounced reduction in PPII propensity near the mutated PXXP motifs, particularly for P72R, which disrupts the two consecutive proline residues in the PRD. This change is accompanied by an increase in turn and bend content (Fig. 8, *a*). Minimum distance maps indicate mutation-specific local effects (Fig. 8, *b* and *c*). In P72R, the characteristic steric constraint imposed by consecutive prolines at positions 71–72 is disrupted, increasing local backbone flexibility. In P82L, replacement of a proline within a conserved PXXP motif eliminates a local PPII-stabilizing element. Unlike proline, leucine cannot undergo *cis*-*trans* isomerization (10,11,12), removing an additional source of local conformational heterogeneity present in the WT sequence. Consistent with the mutation of proline to a noncyclic residue, the backbone ϕ/ψ angle distributions exhibit increased flexibility near the mutation sites, reflecting the loss of proline ring-imposed torsional constraints (Fig. S9).

Experimental SAXS profiles are largely unchanged across variants, indicating that these mutations primarily affect local conformational preferences rather than global chain dimensions (Fig. 10, *d*–*f*). NMR observables similarly show no evidence for long-range structural rearrangements (Figs. S13–S15). Changes in chemical shifts at the mutation sites primarily reflect residue identity rather than the formation of new secondary structure.

### Functional implications and broader context

The persistence of PPII-like structure across a wide range of global conformations indicates that the p53 PRD encodes a robust local structural fingerprint that is largely decoupled from overall chain compactness. Such behavior is consistent with the role of IDRs as flexible scaffolds that maintain interaction-competent motifs while sampling diverse global conformations (15). Specifically, the PPII helices emerge as stiff spacers that prevent collapse, separating the transactivation domain from downstream regions, whereas the intervening disordered regions act as flexible hinges.

The modest and localized structural effects of the P72R polymorphism are consistent with its association with altered regulatory outcomes without loss of p53 function (19). In contrast, the P82L mutation disrupts a conserved PXXP motif implicated in SH3-domain binding and apoptotic signaling. Loss of local PPII structure and elimination of *cis*-*trans* proline isomerization provide a mechanistic explanation for the impaired regulatory response associated with this variant (7,21), despite minimal effects on global dimensions. Although the present simulations necessarily overestimate PPII content due to the absence of *cis*-proline conformations, they nonetheless capture key qualitative features of the PRD ensemble and provide a framework for understanding how local structural motifs persist within highly dynamic disordered proteins.

## Conclusions

This work provides a detailed molecular-level characterization of the p53 PRD, revealing how intrinsic disorder and proline-driven conformational preferences jointly shape its structural ensemble. Extensive MD simulations, supported by SAXS, CD, and NMR comparisons, demonstrate that the PRD exists as a highly heterogeneous yet structured ensemble dominated by transient PPII helices interspersed with β-bends and turns. The unusually high proline content, particularly conserved PXXP motifs and consecutive prolines, imposes local rigidity that limits backbone collapse while preserving global disorder.

By modeling the PRD both as an isolated IDP and as an internally constrained disordered region, we show that its transient secondary-structure fingerprint is remarkably robust to global spatial restraints. End-to-end restrained simulations reproduce experimental SAXS data more accurately while maintaining local PPII propensities. This supports the view that the PRD functions not merely as a flexible tether, but as a semirigid spacer within full-length p53, where PPII helices provide structural stiffness, and the intervening disordered regions act as flexible hinges. This architecture enables conformational flexibility while preserving interaction-competent motifs required for signaling and regulatory binding.

Analysis of the P72R and P82L variants highlights the functional importance of proline-mediated structure. The P72R polymorphism disrupts local rigidity by removing consecutive prolines, leading to increased flexibility without major global effects. In contrast, P82L abolishes a conserved PXXP motif and its associated PPII structure. This provides a mechanistic explanation for impaired PIN1-dependent regulation and altered apoptotic signaling observed in this variant (7,21). These mutation-specific effects underscore how subtle local changes within IDRs can have outsized functional consequences.

More broadly, this study illustrates how proline patterning, transient PPII structure, and *cis*-*trans* isomerization collectively modulate the conformational landscapes of proline-rich IDRs. Although conventional MD simulations necessarily overestimate PPII content due to limited sampling of *cis*-proline states (10,11), the present results capture key qualitative features of the PRD ensemble and establish a framework for integrating experimental data with tailored simulation strategies. This approach should be broadly applicable to the study of flexible, proline-rich regions involved in signaling, molecular recognition, and disease.

## Data and code availability

The data sets measured, generated, and/or analyzed during the current study are available in the Zenodo repository (https://doi.org/10.5281/zenodo.19207053) as well as in the Small Angle Scattering Biological Data Bank: SASDY42, SASDY52, SASDY62, SASDY72, SASDY82, and SASDY92.

• https://www.sasbdb.org/data/SASDY42/htgomv0ppp
• https://www.sasbdb.org/data/SASDY52/m4yyaf88gg
• https://www.sasbdb.org/data/SASDY62/xoqu598yni
• https://www.sasbdb.org/data/SASDY72/45yw2vcyby
• https://www.sasbdb.org/data/SASDY82/1pu1c30y7o

## Acknowledgments

We acknowledge financial support from the Royal Society of Physiography in Lund, and the Carl Trygger Foundation, in Sweden. The simulations were enabled by resources provided by the National Academic Infrastructure for Supercomputing in Sweden, NAISS, and partially funded by the 10.13039/501100004359Swedish Research Council through grant agreement no. 2022-06725 and resources provided by the Swedish National Infrastructure for Computing, SNIC, at the Center for Scientific and Technical Computing at Lund University, LUNARC, Sweden, partially funded by the 10.13039/501100004359Swedish Research Council through grant agreement no. 2018-05973. We acknowledge the 10.13039/501100001671European Synchrotron Radiation Facility, ESRF, France, for beamtime MX-2673 (https://doi.org/10.15151/ESRF-ES-2001352989) and MX-2757 (https://doi.org/10.15151/ESRF-ES-2227803777) as well as the Center for Storage Ring Facilities in Aarhus, ISA, in Denmark. This project has received funding from the European Union’s Horizon 2020 research and innovation programme under the grant agreement no. 101004806, MOSBRI-2021-29. We acknowledge our local contact, Nykola Jones, for the help provided during the beamtime at ISA.

## Author contributions

A.B.: conceptualization, methodology, validation, formal analysis, investigation, visualization, writing – original draft, review, and editing. M.B.: methodology, investigation, writing – original draft, review, and editing. H.F.: methodology, investigation, writing – original draft, review, and editing. M.S.: conceptualization, methodology, validation, resources, data curation, writing – original draft, review, editing, supervision, project administration, and funding acquisition.

## Declaration of interests

The authors declare no competing interests.

## Supporting material

Document S1. Figures S1–S16 and Table S1

Document S2. Article plus Supporting Material
