# Supplementary material for: Conformational flexibility and transient structure of the proline-rich domain in p53
Source: Biophys J. 2026 Mar 14;125(8):1914–25. doi: 10.1016/j.bpj.2026.03.024 (PMC13351842; doi:10.1016/j.bpj.2026.03.024)
Supplement: Document S1. Figures S1–S16 and Table S1 [file mmc1.pdf]

Biophysical Journal, Volume *125*

Supplemental information

Conformational flexibility and transient structure of the proline-rich domain in p53

Agnes Berggren, Michael Bakker, Hayden Fisher, and Marie Skepö

Article

Conformational Flexibility and Transient Structure of the Proline-Rich Domain in p53

Agnes Berggren[1,2], Michael Bakker[3], Hayden Fisher[4], and Marie Skepö[1,2,*]

[1]Division of Computational Chemistry, Department of Chemistry, Lund University, P.O. Box 124, 22100 Lund, Sweden
[2]NanoLund, Lund University, P.O. Box 118, 22100 Lund, Sweden
[3]Faculty of Pharmacy in Hradec Králové, Charles University, Akademika Heyrovského 1203/8, 500 05 Hradec Králové, Czech Republic
[4]European Synchrotron Radiation Facility, Cedex 9, 38043 Grenoble, France
[*]Correspondence: marie.skepo@compchem.lu.se

SUPPLEMENTARY INFORMATION

Table 1: Table showing the average DSSP over all residues in the proline-rich domain from the simulations. WT is the unrestrained wild-type simulations, for Pro82 in the *trans* and *cis* conformation, respectively. P72R and P82L are point mutations were residues P72 and P82, respectively, have been substituted. R_{ee} is the fixed end-to-end distance that has been restrained in the simulations.

**DSSP**	**WT *trans***	**WT *cis***	R_{ee}=10.5 Å	R_{ee}=21.9 Å	R_{ee}=30.5 Å	R_{ee}=40.7 Å	R_{ee}=51.2 Å	**P72R**	**P82L**
β-bend	14.4	18.2	16.7	17.7	16.4	18.1	16.8	15.0	16.1
Turn	5.3	5.1	6.4	6.6	4.8	5.0	3.5	5.3	5.9
Polyproline II-helix	31.1	28.1	26.4	25.1	28.1	26.6	28.1	27.7	27.8
π-helix	0.0	2.8	4.3	0.0	0.0	0.0	0.0	0.0	0.0
3_{10}-helix	0.8	0.8	0.4	0.6	0.2	0.3	0.6	0.9	0.6
β-strand	0.2	0.2	1.3	1.5	0.1	0.1	0.0	0.4	0.1
β-bridge	0.4	0.6	1.2	0.8	0.7	0.5	0.3	0.9	0.8
α-helix	0.2	0.0	0.0	0.0	0.0	0.0	0.3	0.0	0.1
Unordered	45.1	44.4	45.5	45.6	47.5	47.3	48.2	47.2	46.2

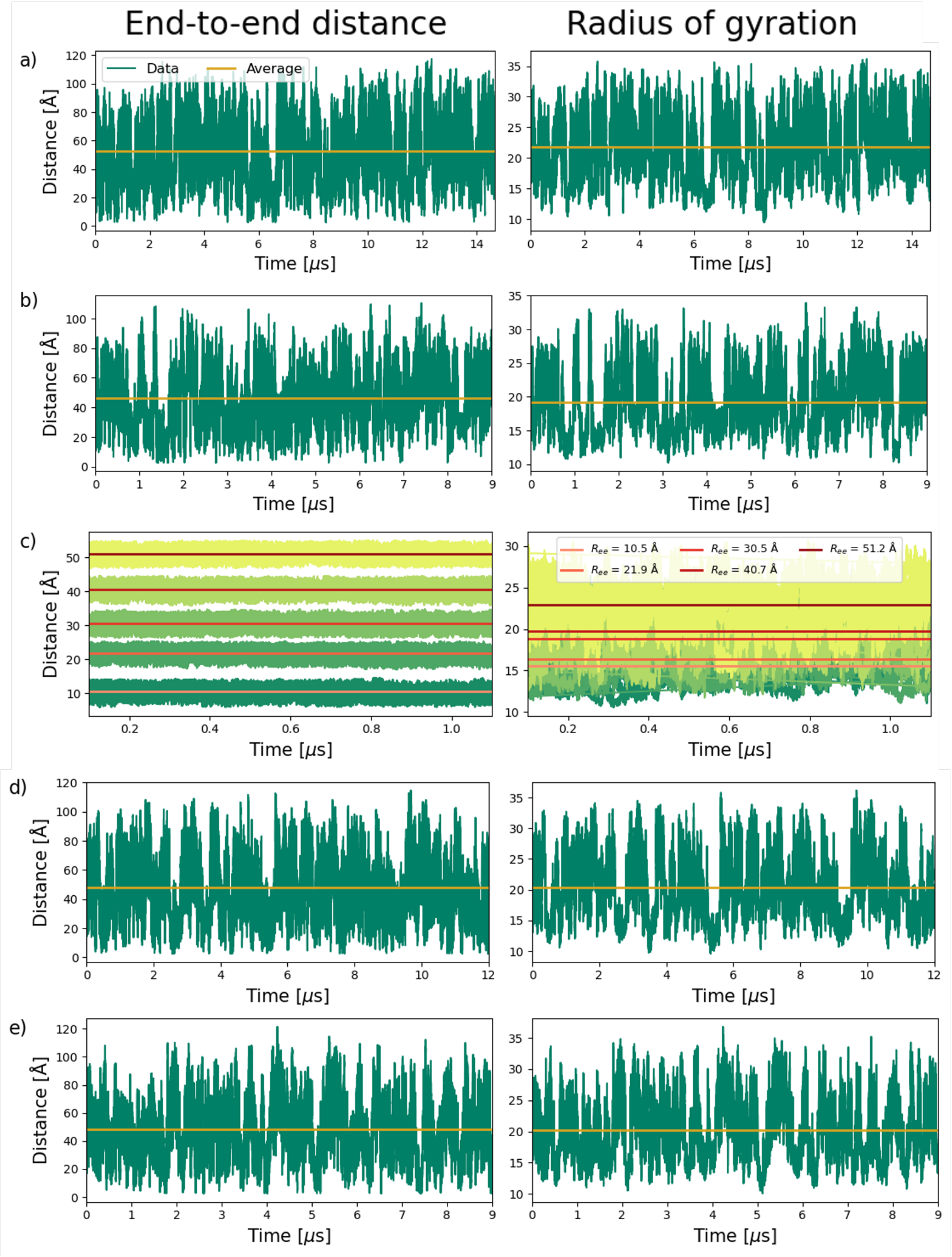

Figure 1: Figure shows the variation of the end-to-end distance and radius of gyration for each frame during the a) unrestrained WT, b) unrestrained cis-Pro82 WT, c) restrained WT (R_{ee}=10-50 Å), d) P72R, and e) P82L simulations.

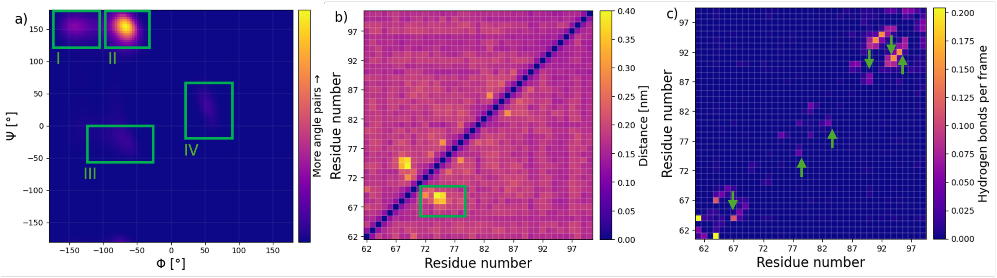

Figure 2: a) Ramachandran plot showing the Phi, ϕ, and Psi, ψ, angles of the protein backbone. Structures known to occur in the Ramachandran regions are I) β-strands, II) PPII helices, III) 3_{10}- and right-handed α-helices, and IV) left-handed α-helices. b) Minimum distance map shows the shortest distance between any pair of amino acid residues, and c) Average number of hydrogen bonds between residues within the protein domain.

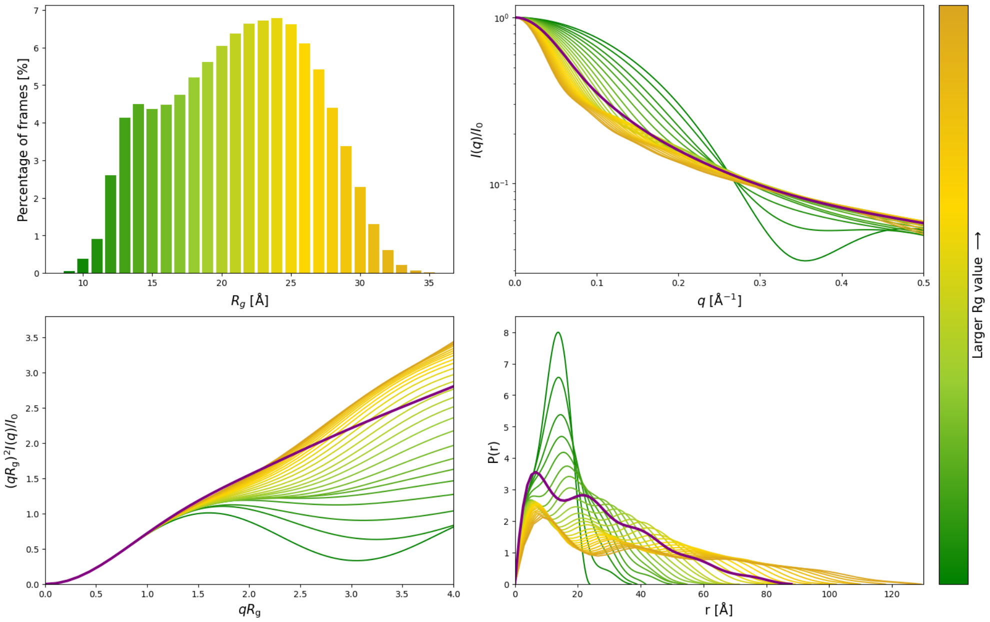

Figure 3: Figure is showing the SAXS intensity plot, Kratky plot and pair distance distribution plot for WT 10 mM NaCl simulations (black). The conformational ensemble has been decomposed based on the radius of gyration, R_g. This means that the individual frames of the simulation have been organised into groups based on their R_g (see top left figure) and the SAXS curves calculated within each group. The plots have been normalised in I_0 for easy comparison.

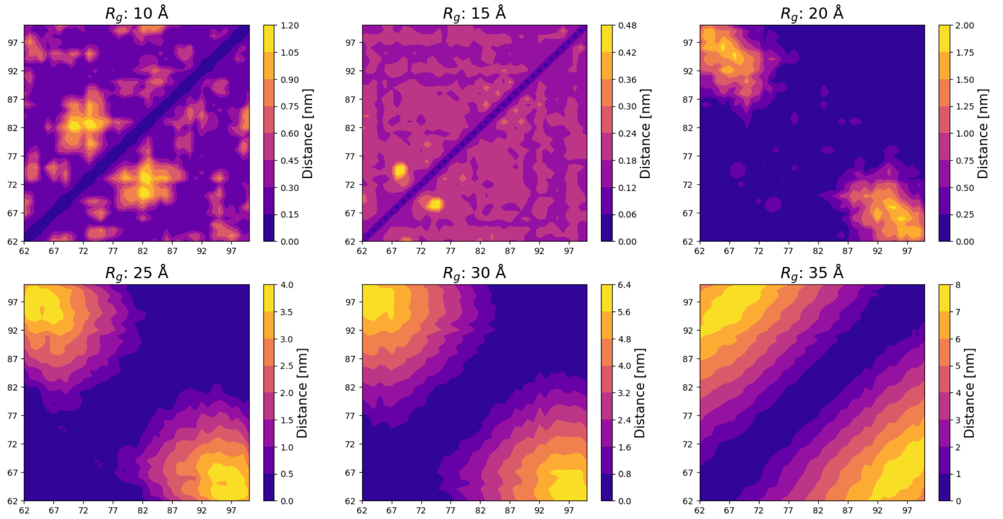

Figure 4: Figure is showing the minimum distance between any pair of amino acid residues for the simulation of the unrestrained wild-type proline-rich domain. The conformational ensemble has been decomposed based on the radius of gyration, R_g. This means that the individual frames of the simulation have been organised into groups based on their R_g and analysed individually, with the results for R_g 10, 15, 20, 25, 30, and 35 Å being presented in the figure.

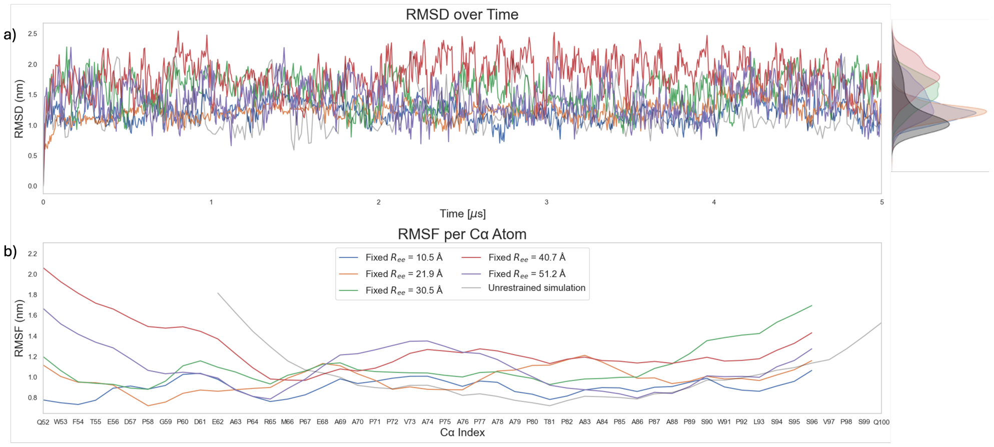

Figure 5: a) Root mean square deviation (RMSD) of the atomic positions throughout the molecular dynamics simulations, plotted as a function of simulation time. b) Root mean square fluctuation (RMSF) of atomic positions for each amino acid residue. This is shown for both unrestrained and restrained simulations, where R_{ee} is the fixed end-to-end distance.

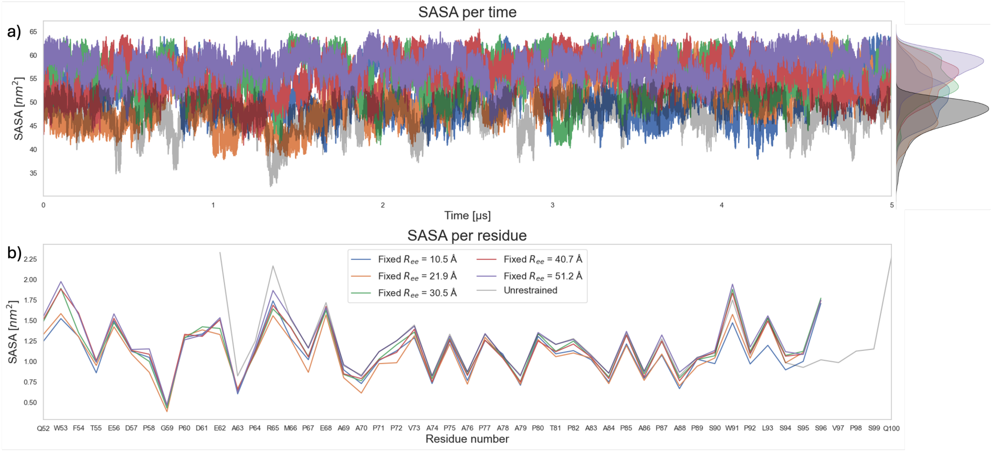

Figure 6: Solvent accessible surface area (SASA), plotted as a function of simulation time (a) and per residue (b). This is shown for both unrestrained and restrained simulations, where R_{ee} is the fixed end-to-end distance.

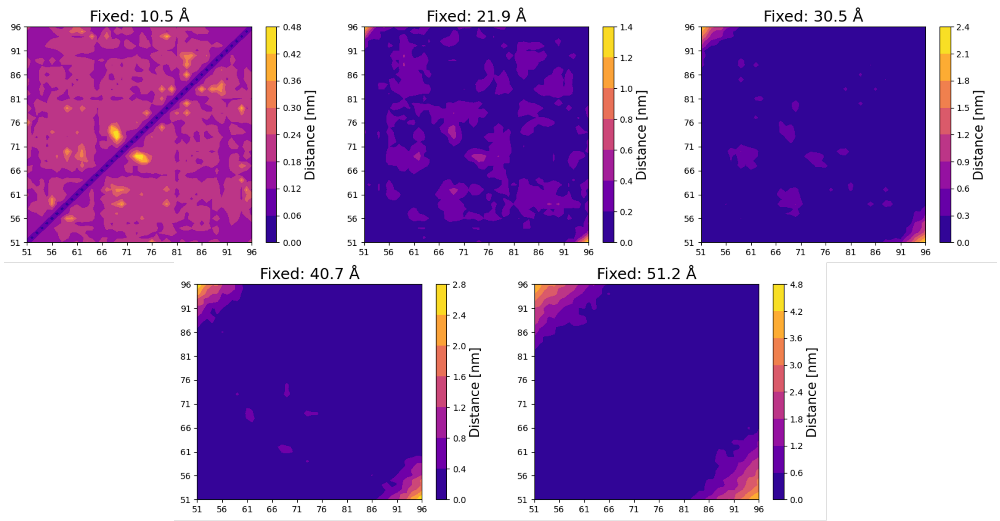

Figure 7: Figure is showing the minimum distance between any pair of amino acid residues for the simulations of the restrained wild-type proline-rich domain. End-to-end distance is fixed for the restrained simulations.

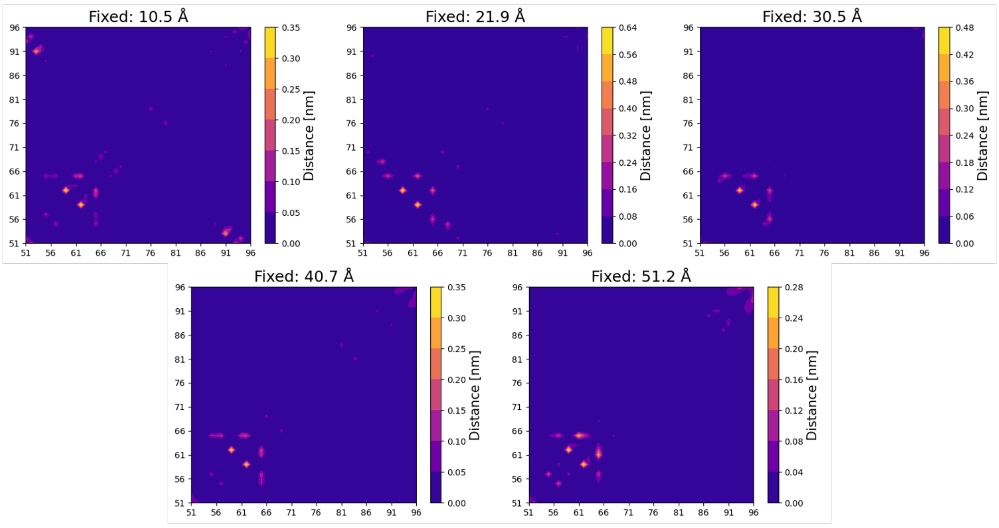

Figure 8: Figure is showing the average number of hydrogen bonds between any pair of amino acid residues for the simulations of the restrained wild-type proline-rich domain. End-to-end distance is fixed for the restrained simulations.

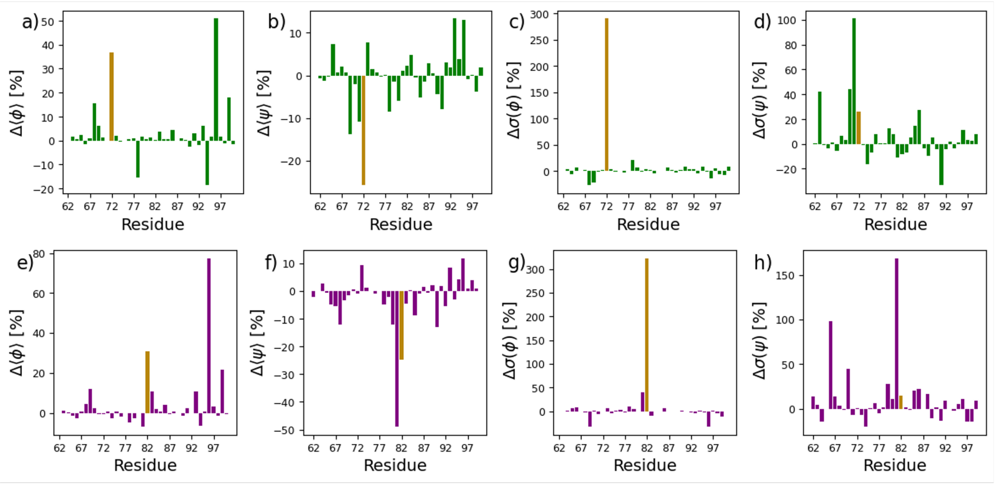

Figure 9: Relative change in the mean Phi, ϕ, and Psi, ψ, angles as a function of residue number for a)-b) P72R (green) and e)-f) P82L (purple), using wild-type (WT) simulations as reference. Relative change in the standard deviation of ϕ and ψ angles as a function of residue number for c)-d) P72R (green) and g)-h) P82L (purple), using wild-type simulations as reference. The yellow bar indicates the mutated residue.

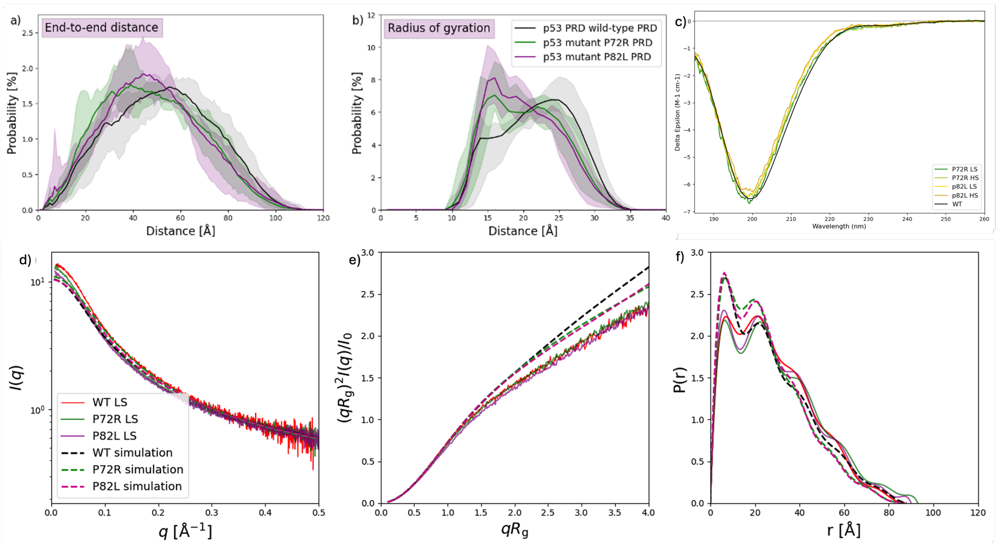

Figure 10: Figure shows the distribution of the end-to-end distance (a) and radius of gyration (b) during the entire simulations for wild-type proline-rich domain and two mutants P72R and P82L. The shaded area shows the deviation from the average for the five separate replicates. The lines are the average. C) Delta epsilon ($\Delta\epsilon$) from circular dichroism for P72R and P82L, across two salt concentrations 10 mM (LS) and 150 mM (HS). WT is the $\Delta\epsilon$ from the wild-type PRD. Small-angle X-ray scattering results from experiments and simulations for wild-type (WT) and mutant (P72R and P82L) proline-rich domain. d) Form factor comparing the scattering vector to the normalised intensity, e) Kratky plot, and f) Pair distance distribution function. All SAXS data from simulations and experiments were performed at low salt concentration (10 mM NaCl).

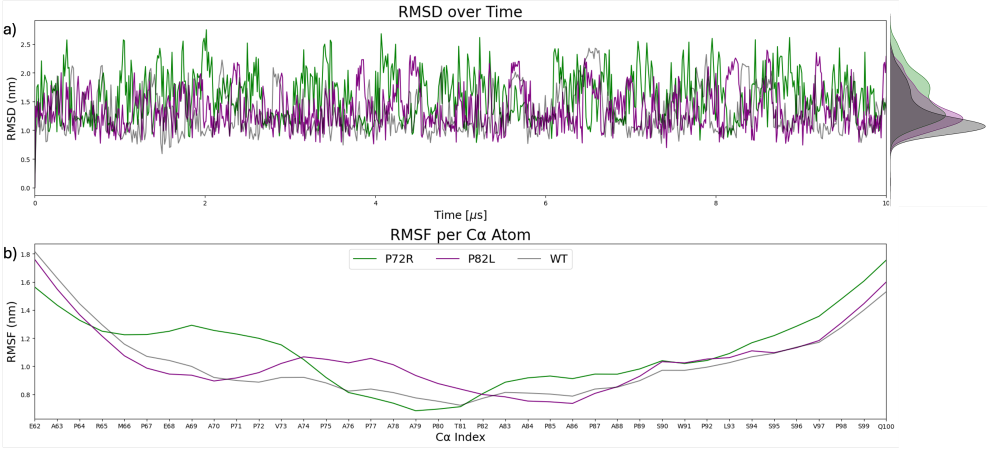

Figure 11: a) Root mean square deviation (RMSD) of the atomic positions throughout the molecular dynamics simulations, plotted as a function of simulation time. b) Root mean square fluctuation (RMSF) of atomic positions for each amino acid residue. This is shown for P72R, P82L and WT variants.

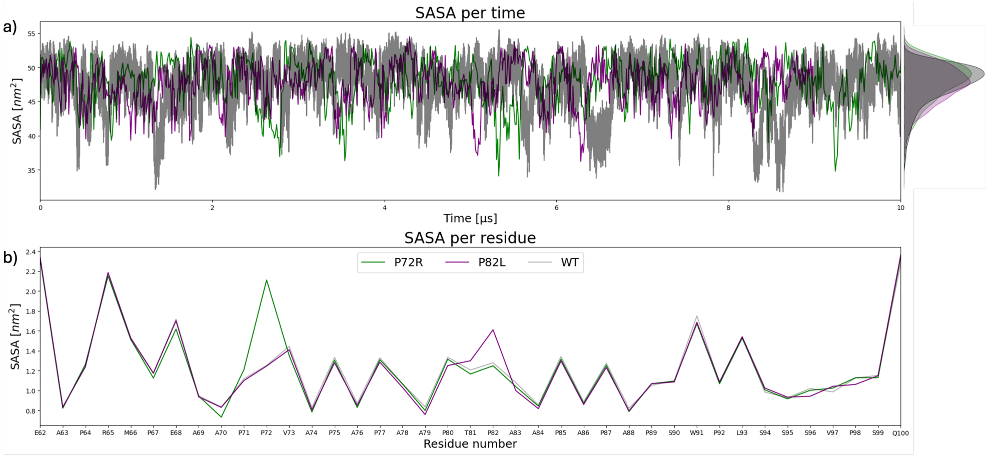

Figure 12: Solvent accessible surface area (SASA), plotted as a function of simulation time (a) and per residue (b). This is shown for P72R, P82L and WT variants.

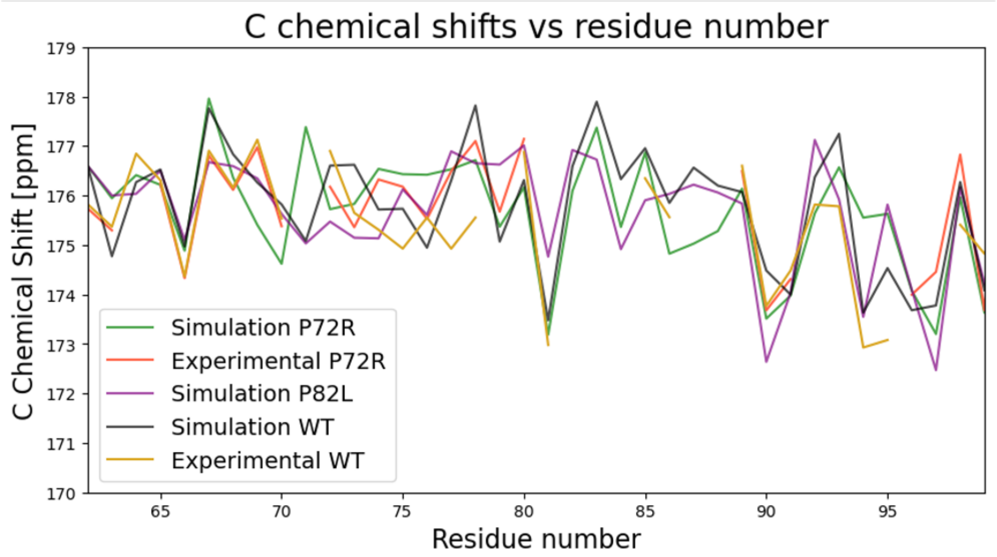

Figure 13: C chemical shifts per residue are presented for the simulated variants P72R, P82L, and WT. These are compared to experimental chemical shifts obtained from the Biological Magnetic Resonance Data Bank (BMRB) using entries 51984 (P72R) and 50960 (WT).

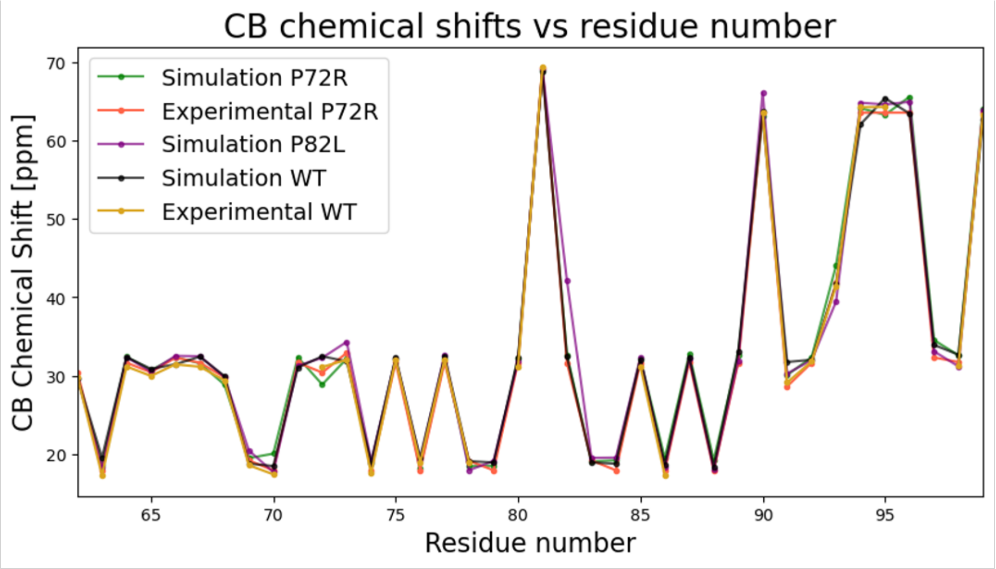

Figure 14: CB chemical shifts per residue are presented for the simulated variants P72R, P82L, and WT. These are compared to experimental chemical shifts obtained from the Biological Magnetic Resonance Data Bank (BMRB) using entries 51984 (P72R) and 50960 (WT).

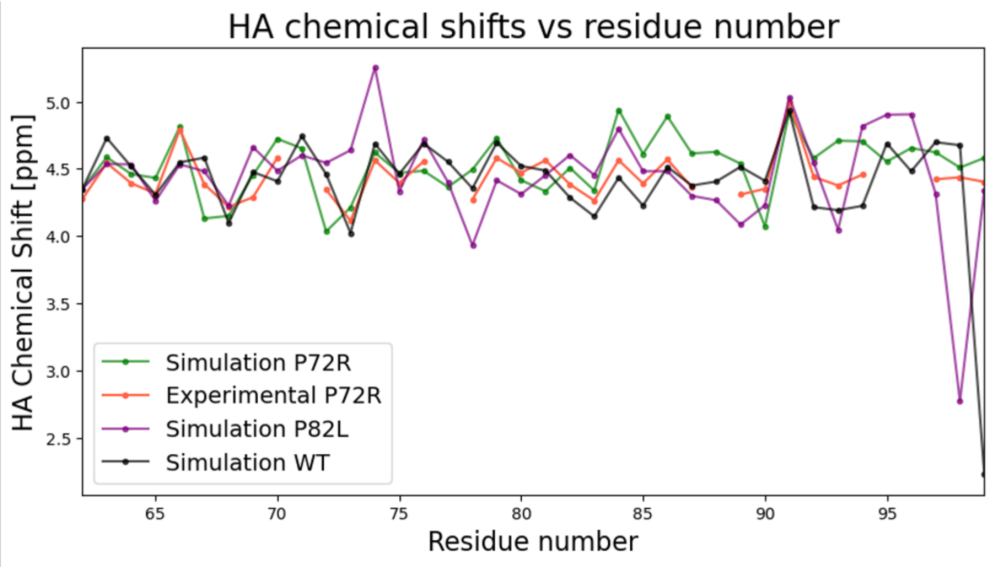

Figure 15: HA chemical shifts per residue are presented for the simulated variants P72R, P82L, and WT. These are compared to experimental chemical shifts obtained from the Biological Magnetic Resonance Data Bank (BMRB) using entry 51984 (P72R).

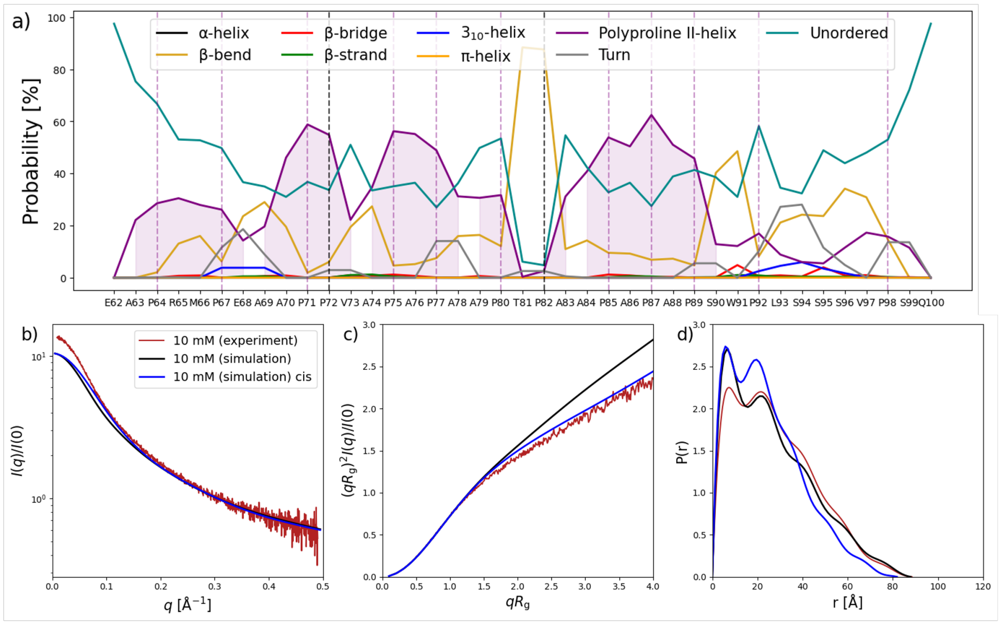

Figure 16: a) Residue-wise secondary structure probability. The PXXP (Proline-any-any-Proline) motifs are underlined, and all proline residues are marked with vertical dashed lines. The black dashed lines are Pro72 and Pro82. The five polyproline II (PPII) helical peaks are shaded for emphasis. b) Form factor comparing the scattering vector to the normalised intensity, c) Kratky plot, and d) Pair distance distribution function. All simulations and the experiment were performed at low salt concentration (10 mM NaCl).
